# Open-Top Patterned Hydrogel-Laden 3D Glioma Cell Cultures for Creation of Dynamic Chemotactic Gradients to Direct Cell Migration

**DOI:** 10.1021/acsbiomaterials.4c00041

**Published:** 2024-04-23

**Authors:** Aditya Rane, Steven Tate, Jenna L. Sumey, Qing Zhong, Hui Zong, Benjamin Purow, Steven R. Caliari, Nathan S. Swami

**Affiliations:** † Chemistry, University of Virginia, Charlottesville, Virginia 22904, United States; ‡ Electrical and Computer Engineering, University of Virginia, Charlottesville, Virginia 22904, United States; § Chemical Engineering, University of Virginia, Charlottesville, Virginia 22904, United States; ∥ Neurology, School of Medicine, University of Virginia, Charlottesville, Virginia 22903, United States; ⊥ Microbiology, Immunology & Cancer Biology, School of Medicine, University of Virginia, Charlottesville, Virginia 22903, United States; ∇ Biomedical Engineering, University of Virginia, Charlottesville, Virginia 22904, United States

**Keywords:** hydrogel, microfluidics, tumor microenvironment, cell migration, glioma

## Abstract

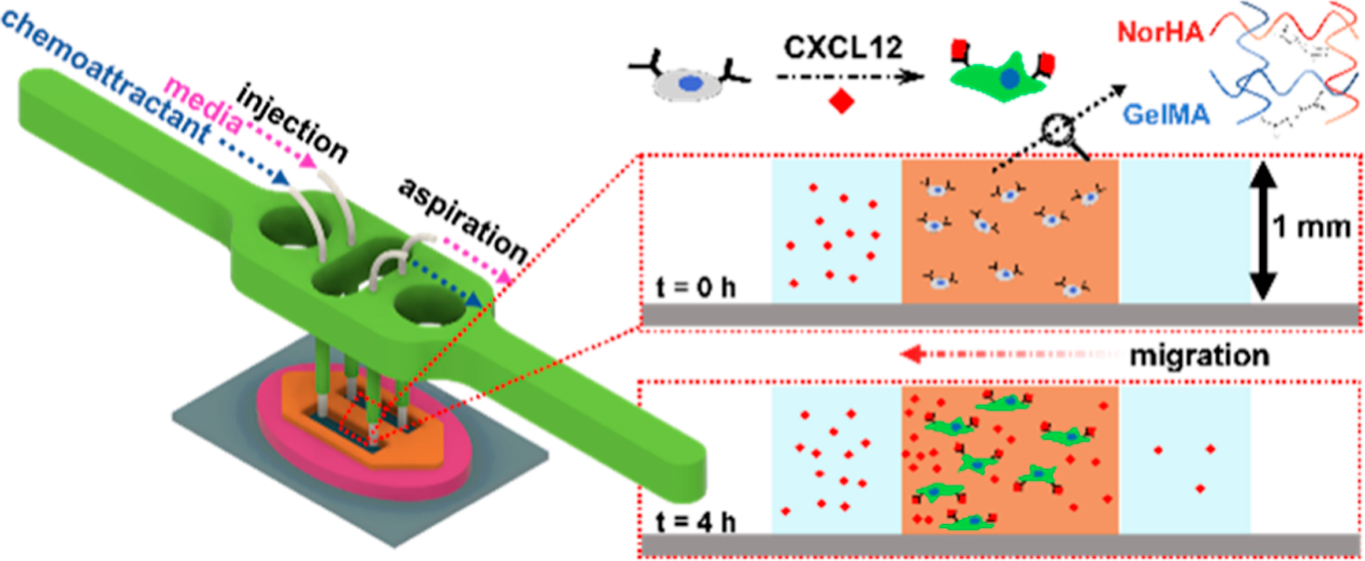

The laminar flow profiles in microfluidic systems coupled to rapid diffusion at flow streamlines have been widely utilized to create well-controlled chemical gradients in cell cultures for spatially directing cell migration. However, within hydrogel-based closed microfluidic systems of limited depth (≤0.1 mm), the biomechanical cues for the cell culture are dominated by cell interactions with channel surfaces rather than with the hydrogel microenvironment. Also, leaching of poly(dimethylsiloxane) (PDMS) constituents in closed systems and the adsorption of small molecules to PDMS alter chemotactic profiles. To address these limitations, we present the patterning and integration of a PDMS-free open fluidic system, wherein the cell-laden hydrogel directly adjoins longitudinal channels that are designed to create chemotactic gradients across the 3D culture width, while maintaining uniformity across its ∼1 mm depth to enhance cell–biomaterial interactions. This hydrogel-based open fluidic system is assessed for its ability to direct migration of U87 glioma cells using a hybrid hydrogel that includes hyaluronic acid (HA) to mimic the brain tumor microenvironment and gelatin methacrylate (GelMA) to offer the adhesion motifs for promoting cell migration. Chemotactic gradients to induce cell migration across the hydrogel width are assessed using the chemokine CXCL12, and its inhibition by AMD3100 is validated. This open-top hydrogel-based fluidic system to deliver chemoattractant cues over square-centimeter-scale areas and millimeter-scale depths can potentially serve as a robust screening platform to assess emerging glioma models and chemotherapeutic agents to eradicate them.

Glioblastoma (GBM) is the most common and aggressive type of primary brain cancer in adults^1^ that remains incurable and recurs frequently,^2^ highlighting the need for *in vitro* patient-specific models that can predict disease outcomes. Recent work has correlated the infiltrative nature of the disease to glioma cell migration characteristics,^3^ but recapitulation of the chemical and biomechanical cues that affect cell migration in the tumor microenvironment (TME) remains a major challenge within these models.^4^ Based on hyaluronan, which is a primary extracellular matrix component in the brain, hyaluronic acid hydrogel networks are known to induce dose-dependent alterations to markers of glioma malignancy.^5^ The inclusion of hyaluronic acid-based hydrogel as a matrix, with soluble CXCL12 as a chemoattractant for CXCR4-expressing GBM cells^6^ and with chemotherapeutic agents to eradicate them,^7^ is being investigated as a less invasive and more targeted pathway to remove residual glioma cells from the TME. To complement prior work on coupling laminar flow profiles to rapid diffusion at flow streamlines in closed microfluidic systems,^8^ we present an open-top microfluidic platform to create chemotactic gradients for spatiotemporal control of glioma cell migration in a patterned hydrogel of millimeter-scale depth that is designed to enhance cell–biomaterial interactions.

Pressure-based microfluidic flow control in closed microchannels for cell culture within poly(dimethylsiloxane) (PDMS) backing layers on one side to enable fluidic access and bonded to a glass coverslip to enable access to live-cell imaging, is commonly used to investigate cellular processes. However, to create 3D cultures with biomechanical cues from the hydrogel-based cell microenvironment, without being limited by cell–cell interactions or cell–surface interactions, the cultures must be patterned over several cell layers (∼mm-scale depths) between the flows that deliver the chemotactic cues. The chief challenge to maintaining such 3D cultures in closed microfluidic systems over several days for directing cell migration^9^ is the leaching of PDMS monomers into the culture medium, which affects cell proliferation, cell adhesion, and differentiation of cells in pharmacokinetic studies.^10−12^ Furthermore, the adsorption of small hydrophobic molecules by PDMS over the long term of the cell culture affects their transport to the culture, thereby altering dose response and drug gradients.^13,14^ Also, alterations in oxygen permeability with PDMS bonding^15^ and its high permeability to water vapor^16,17^ can cause culture media evaporation, drying, and bubble formation that are detrimental to the establishment of chemotactic gradients.^18^ Alternate substrates for 3D cell culture, such as PMMA, COC, and adhesive tapes, usually involve cumbersome fabrication and assembly steps that limit rapid prototyping of device designs and assays.^19,20^ The availability of open-top hydrogel-laden 3D cultures devoid of PDMS interfaces would mitigate many of these limitations, but current reports^21^ do not yet integrate fluidic control operations for dynamic modulation of gradients that provide chemotactic cues to direct cell migration.

The patterning of cell-laden hydrogels for 3D culture in closed microfluidic systems is usually accomplished with arrays of microposts or pillars that use the surface tension of the hydrogel material to confine it between the adjoining media channels. To prevent the hydrogel from spilling into the adjoining fluidic channel, the injection pressure must be carefully controlled,^22−24^ which depends on the viscosity, wettability, and other material properties of the hydrogel. High-aspect-ratio microfabrication is needed to create posts that extend several micrometers in the lateral scale and up to millimeters in the depth scale to contain cell-laden hydrogels for 3D culture. This can lead to discontinuities in the interface between the hydrogel and the perfused medium, thereby subjecting the cells to altered biochemical cues.^22^ Recent approaches to create continuous interfaces of the hydrogel to the adjoining fluid in closed channels have emerged, such as the use of phase guides, stepped height channels, recoverable elastic barriers, and alignment of core–shells, but these require multilayer fabrication and assembly.^23−27^ Rail-based capillary-pinning approaches for the patterning of open-top 3D cell cultures have been reported^21,28,29^ but are static and without the fluidic control needed to deliver chemical gradients for dose/drug response assays or directed migration studies. Microfluidic probes (MFPs) to deliver gradients to open-top cell cultures by using injection and aspiration flows^30−38^ require careful optimization of the geometry and flow rates of the probes.^35−40^ Also, these are impractical for use in patterned 3D hydrogel cultures due to limited depth control of the confined fluid and depletion of the medium that immerses the 3D culture. To address these issues, we present a PDMS-free open microfluidic system (Figure 1A) integrating the patterned cell-laden hydrogel (∼1 mm depth) with adjoining longitudinal microchannels for dynamic flow control to create chemotactic gradients across the 3D culture width to direct glioma cell migration.

**Figure 1:**
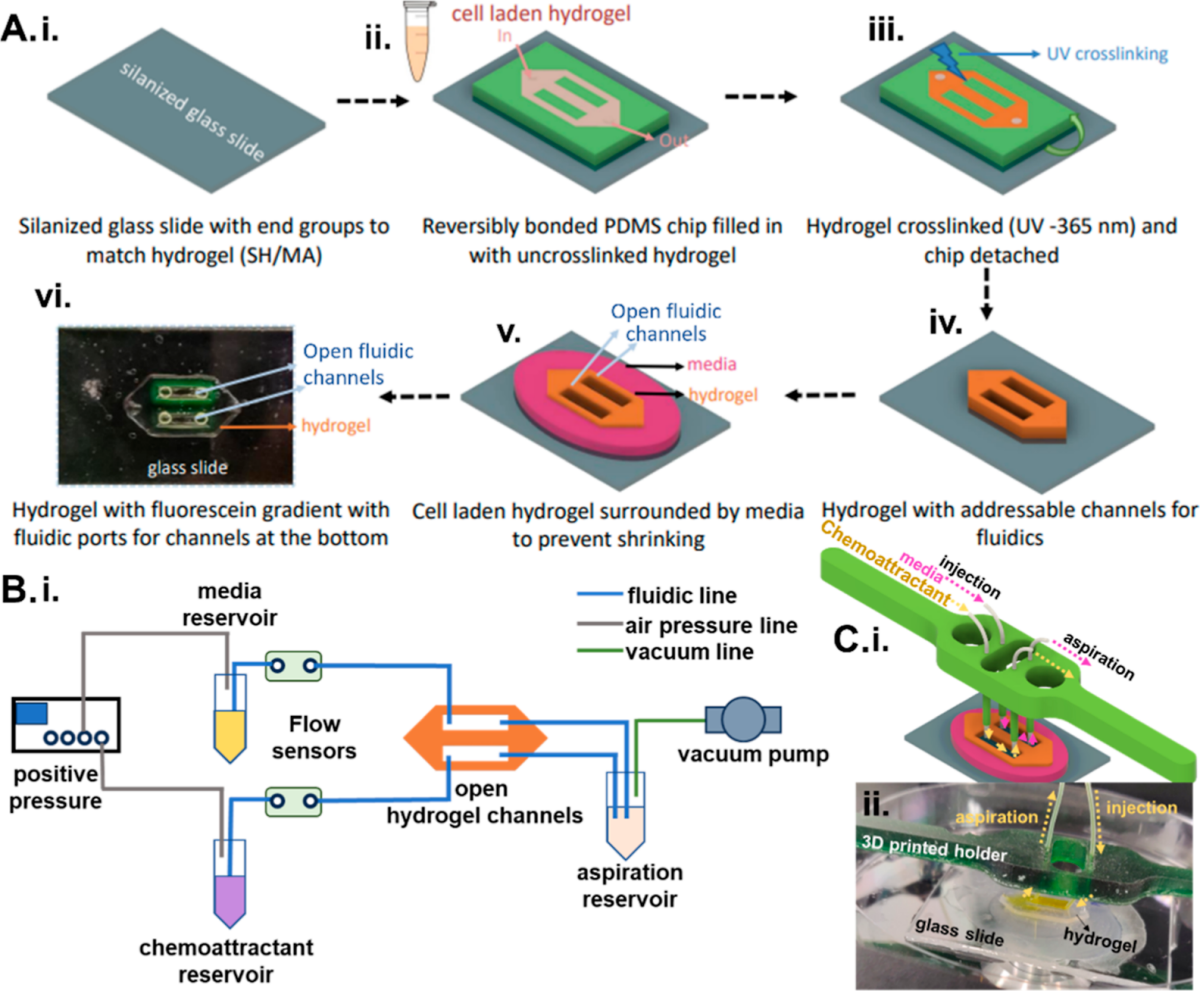
(A) Patterned cell-laden hydrogel adjoining fluidic channels. (i) A silanized glass substrate treated for adhesion to the cross-linked hydrogel is (ii) reversibly bonded to a PDMS mold that is then filled with the cell-laden hydrogel and (iii) photo-cross-linked to create the patterned hydrogel on glass. (iv) The PDMS mold is removed to leave open fluidic channels that directly adjoin the patterned hydrogel. (v) The structure is surrounded with culture medium to maintain cell viability and prevent hydrogel shrinkage. (vi) An example of the patterned hydrogel with addressable open fluidic channels through which a FITC gradient was established. (B) (i) Microfluidic flow control setup. (C) (i) 3D-printed holder for fluidic integration with the patterned hydrogel and (ii) image of the patterned hydrogel with tubing to the 3D-printed holder and channel with yellow dye.

While a reversibly bonded PDMS mold to glass is used as a receptacle to fill in and cure the hydrogel to enable its patterning, this PDMS layer is carefully detached after hydrogel curing, leaving behind only the patterned hydrogel layer adjoining the addressable fluidic channels. Another essential feature of our design is the integration of flow control (Figure 1B) over centimeter-scale lengths in the longitudinal open fluidic channels to deliver the culture medium and remove the waste products, while enabling dynamic modulation of the gradient of chemotactic molecules across the cell-laden hydrogel. This is accomplished through injection flow at one end and aspiration flow on the other end, which are placed in a 3D-printed construct to adjust the height of the microfluidic probes at the injection side to be below the hydrogel height level (Figure 1C(i,ii)), and the aspiration tubing to be just above the hydrogel height level, with surface tension confining the culture medium within the channels over the flow length. Rather than perfusing the culture with a peristaltic pump that creates pulsatile flow, which is difficult to monitor and control over the 48 h culture period, a pressure pump is used to deliver injection fluid at a continuous flow rate, and a vacuum line is used for the aspiration flow. In this manner, a set of integrated flow sensors can continually monitor any alterations and correct them by modulating the injection or aspiration flow rates, thereby maintaining an open cell culture within an incubator jacket, without drying of the hydrogel. Using a flow rate of 7.5 μL/min, which is close to the level reported for interstitial flow in brain tissues,^41−43^ we present the ability to tune chemical gradients to the cells within the patterned hydrogel culture. This open-top system is validated using a patterned culture of U87 glioma cells laden within a hyaluronic acid (HA)–gelatin methacrylate (GelMA) hybrid hydrogel that is optimized to maintain cell viability, while including the adhesive groups necessary for integrin-mediated cell migration. A chemotactic gradient of CXCL12 delivered to the open-top cell-laden hydrogel culture is used to assay migration cues in the presence and absence of AMD3100, an inhibitor to CXCR4-expressing GBM cells. We envision utilization of this open-top integrated system to deliver chemotactic gradients and serve as a drug testing platform for micropatterned cell-laden hydrogel models.

## Results and Discussion

### Optimizing the Hydrogel Composition for Maintaining Cell Viability and Adhesion Motifs for Migration

**Figure 2:**
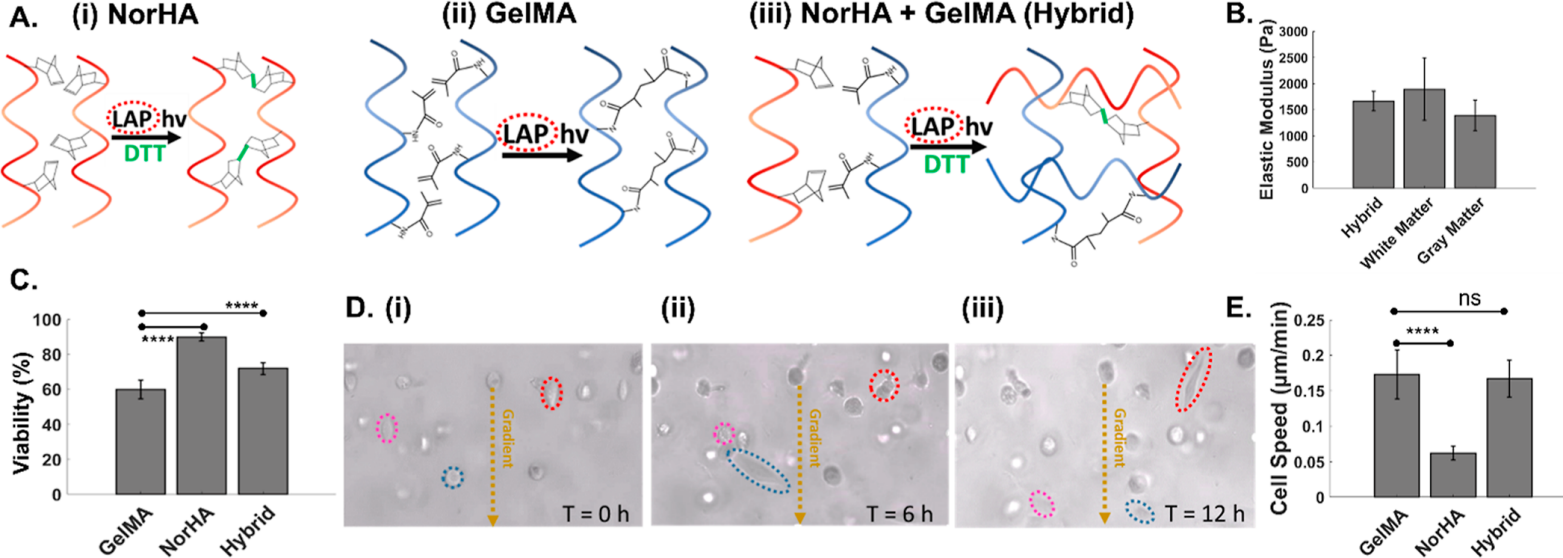
(A) Individual and hybrid hydrogels. Molecular structure before and after cross-linking of (i) norbornene-modified hyaluronic acid (norHA), (ii) gelatin methacrylate (GelMA), and (iii) a hybrid hydrogel of NorHA and GelMA. (B) Young’s modulus of the hybrid hydrogel formulation (2% HA, 1% GelMA) compared to that of brain tissue. The hybrid hydrogel recapitulates the reported stiffness of white and gray matter. (C) Cell viability as a function of hydrogel composition. (D) Cell mobility based on cell migration through the hydrogel at the indicated time points (i)–(iii). (E) Measured cell migration speed as a function of hydrogel composition. Significance was determined by one-way ANOVA with Tukey’s post-hoc test, represented as ns, *p* ≥ 0.05; *, *p* ≤ 0.05; **, *p* ≤ 0.01; ***, *p* ≤ 0.001; ****, *p* ≤ 0.0001.

Hyaluronan is a primary extracellular matrix component in the brain, leading to the interest in utilization of HA hydrogels to mimic the glioma microenvironment. However, while it can interact with cells through cell surface markers such as CD44, it lacks the adhesion motifs necessary for integrin-mediated cell migration.^44^ Hence, we optimized a hybrid photo-cross-linked hydrogel composed of GelMA, which includes adhesive groups such as RGD, with HA to mimic the brain TME. As shown in Figure 2A, this is accomplished using lithium phenyl-2,4,6-trimethylbenzoylphosphinate (LAP) as a common photoinitiator for free-radical-initiated cross-linking of norbornene-modified HA (NorHA) and GelMA hydrogels. While GelMA hydrogels have higher stiffness,^45^ HA hydrogels have been used to mimic the stiffness of native brain tissue.^46^ As shown in Figure 2B, this photopatterned hybrid hydrogel, consisting of 2% GelMA and 1% NorHA, exhibits a Young’s modulus that mimics the brain’s white matter and gray matter components.^47^ Based on propidium iodide staining (Figure 2C), GelMA hydrogels (10%) support U87 cells to a viability level of only 60% over the 48 h culture period, whereas the hybrid hydrogel supports ∼75% cell viability, which is closer to the 90% viability levels observed within 1% NorHA hydrogels.

Differences in pore size between the respective hydrogels can affect cell viability, with 1% NorHA hydrogels reported at a theoretical mesh size of ∼85 nm,^46^ while mesh sizes of 10% GelMA are reported as ∼20 nm.^48^ However, this difference in mesh size, as reported for fully swollen hydrogels, is likely less pronounced in our study, since the hydrogels are confined in the device channels and are somewhat restricted from swelling. Instead, GelMA hydrogels that are formed by chain-growth cross-linking are inhibited by oxygen and require a high concentration of free radicals for initiation of polymerization,^49^ leading to a loss in viability. On the other hand, step-growth-polymerized NorHA hydrogels require lower free radical concentrations and are not inhibited by oxygen,^50^ thereby resulting in faster cross-linking and improved cytocompatibility.^51^ This hybrid hydrogel also supports viability of a 3D culture of highly migratory and malignant oligodendrocyte progenitor cells (OPCs) that are progenitors in glioma^52,53^ (Figure S1). Based on time-lapse images of U87 cell alignment and migration (Figure 2D(i–iii)), the cell speeds in the hybrid hydrogel resemble those observed within the GelMA hydrogel, rather than the HA hydrogel (Figure 2D(iv) and Movie S1). This indicates the optimization of the hybrid hydrogel for its high viability and migration abilities.

### Open-Top Microfluidics for Spatiotemporal Control of Chemotactic Gradients Across Patterned Hydrogel

**Figure 3:**
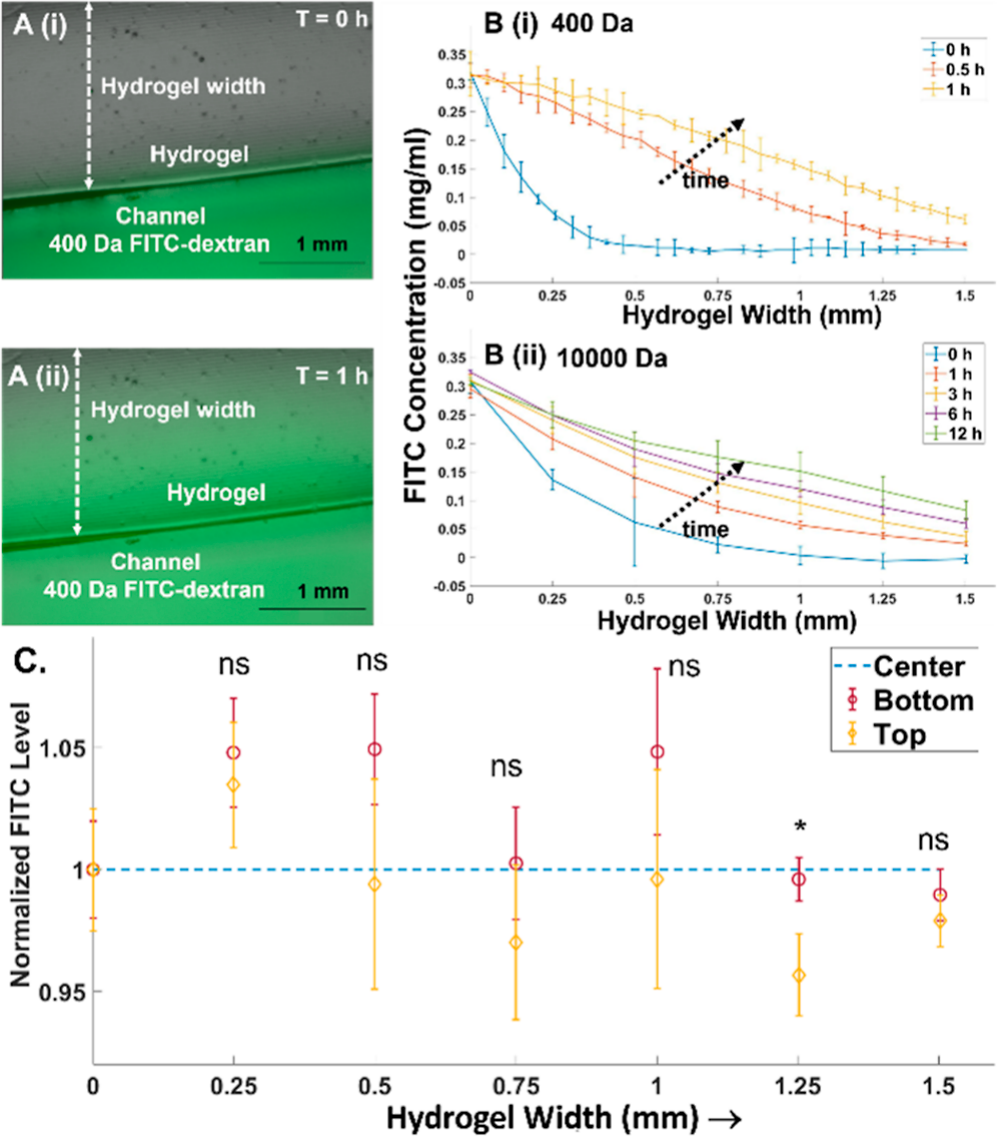
(A) Bright-field and FITC overlay of the gradient across the hydrogel at various time points with 400 Da FITC-dextran at (i) *T* = 0 h and (ii) *T* = 1 h. (B) Temporal development of the gradient across the open hydrogel for (i) 400 Da FITC-dextran, with the gradient developing rapidly to approach steady state within 1 h, and (ii) 10000 Da FITC-dextran, with the gradient developing slowly over 12 h. (C) The chemical gradient develops over the entire hydrogel depth (1 mm) based on the similar FITC levels at the top, bottom, and center of the hydrogel, and its invariance over the hydrogel width at steady state (24 h) for 10000 Da FITC-dextran. Significance is based on one-way ANOVA with Tukey’s post-hoc test, represented as ns, *p* ≥ 0.05; *, *p* ≤ 0.05.

The open-top 3D culture was utilized with longitudinal injection and aspiration flows to deliver and control chemotactic gradients across the patterned hydrogel width. Using fluorescein isothiocyanate (FITC)-labeled dextran (FITC-dextran) with a 400 Da molecular weight to mimic small-molecule drugs, the images (Figure 3A(i,ii)) show development of the gradient across the hydrogel width. Using FITC-dextran with a 10 kDa molecular weight to mimic proteins or cytokines, fluorescence levels measured from time-lapse images show molecular diffusion profiles at different widths across the patterned hydrogel, indicating the ability of the open-top microfluidic system to induce gradual flattening of the initial diffusion profile. It is apparent that the 10 kDa FITC-dextran takes well over 12 h to reach a steady-state profile (Figure 3B(ii)), whereas the 400 Da FITC-dextran reaches deep into the hydrogel within an hour (Figure 3B(i)). This chemotactic gradient can be created across the complete depth of a 1 mm thick hydrogel, as apparent from similar *Z*-stack FITC levels (Figures 3C and S2 and Movie S2) at the top and bottom of the hydrogel (normalized to FITC level at the center). Also, this gradient can be sustained across a large width of the hydrogel (∼2 mm), with an ∼15 mm long uninterrupted and continuous interface between the fluid and hydrogel, whereas prior work had used posts. Hence, the open microfluidic system can create well-defined chemical gradients to viable glioma cells over the long durations needed to assay cell responses (48 h).

### Migration Cues to Glioma Cells in the Patterned Hydrogel Using Open-Top Microfluidics

**Figure 4:**
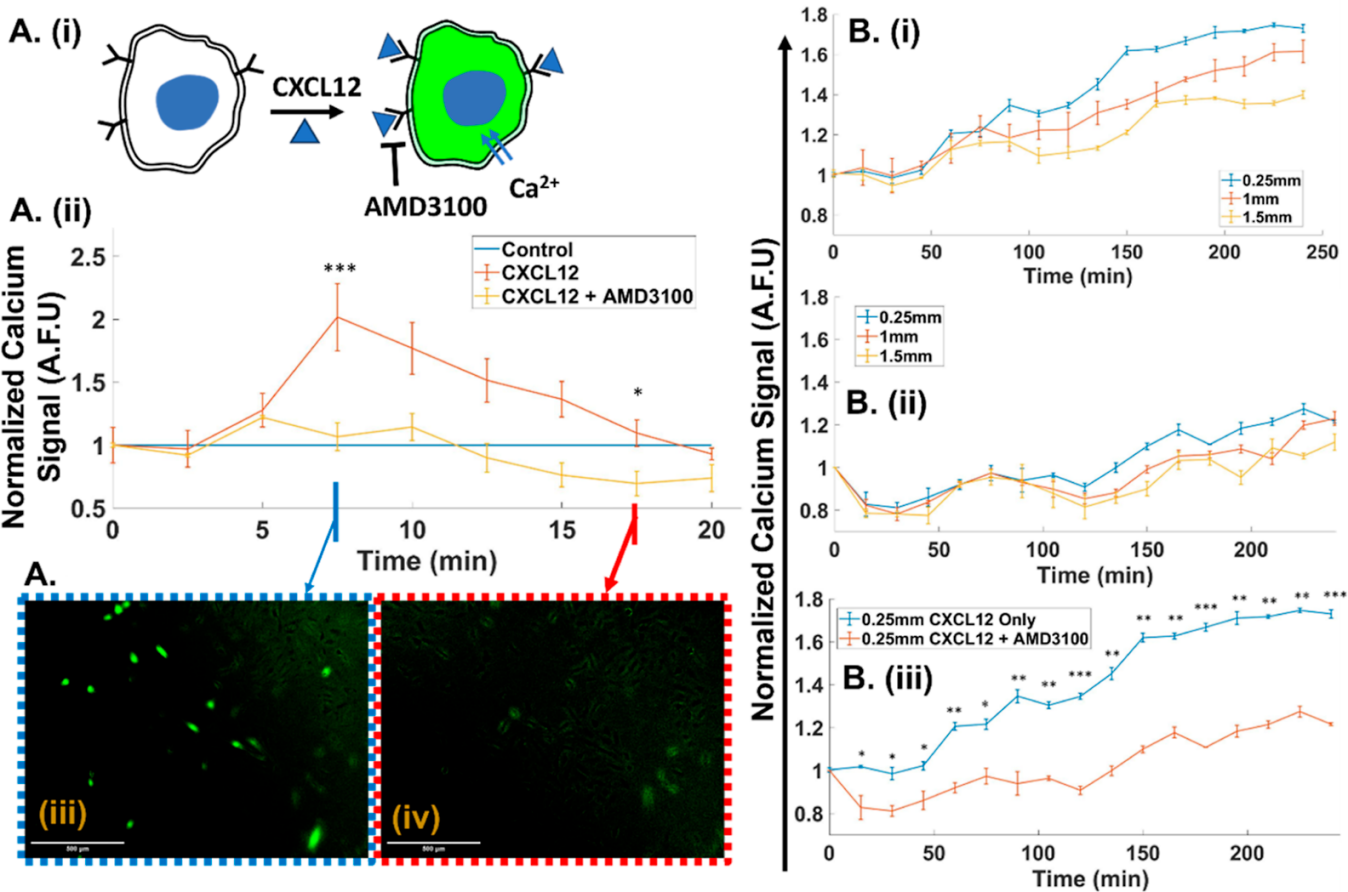
(A) (i) CXCL12 stimulation through calcium ion influx into U87 cells by binding to its receptor to cause fluorescence upon labeling with signaling probe. (ii) CXCL12 stimulation (66 ng/mL) in a static 2D culture causes fluorescence signal rise for ∼5 min, while the minimal signal rise for AMD3100 (1 μg/mL)-pretreated cells validates inhibition of this stimulation. (iii, iv) Fluorescence images with CXCL12 stimulation (scale bar = 500 μm) at indicated time points of (iii) maximum and (iv) baseline or control level with no stimulation. (B) (i) The temporal fluorescence signal from dynamic (7.5 μL/min) 3D cultures of U87 cell-laden hydrogel with 66 ng/mL CXCL12 in the medium rises sharply for cells at a hydrogel width of 0.25 mm, in proximity to the chemoattractant channel boundary, while widths farther from this boundary show less signal increase. (ii) The signal at the same hydrogel widths is inhibited after pretreatment with AMD3100 (1 μg/mL). (iii) Comparison of CXCL12-stimulated cells at the hydrogel width of 0.25 mm, without and with AMD3100 pretreatment, validates inhibition of CXCL12 stimulation. Significance of the differences between cells in the hydrogel under stimulation vs inhibition at the same time point were determined by one-way ANOVA with Tukey’s post-hoc test: *, *p* ≤ 0.05; **, *p* ≤ 0.01; ***, *p* ≤ 0.001; ****, *p* ≤ 0.0001.

To assess the ability of the open-top microfluidic system to deliver chemotactic gradients for cues to cells across the hydrogel width, we utilized the chemokine: CXCL12, which induces migration of CXCR4-expressing glioma cells, and AMD3100, which inhibits this signaling pathway.^54^ As shown in the schematic in Figure 4A(i), gradients of CXCL12 induce calcium influx into the cell upon binding to its cell membrane receptor. Hence, glioma cells labeled with a calcium signaling probe that fluoresces upon calcium binding were used to quantify effect of the chemotactic gradient on cells across the hydrogel width. Fluorescence image analysis of a static culture of glioma cells was used to determine the CXCL12 level in the medium that is needed for signal rise above the baseline. Similarly, inhibition of CXCL12 stimulation at this level was validated using cells pretreated with 1 μg/mL AMD3100.^54^ Based on fluorescence signal plots (Figure 4A(ii)) and images (Figure 4A(iii,iv)), we infer that 66 ng/mL CXCL12 in the medium is sufficient to cause signal rise within 5 min of stimulation, and this signal rise is effectively inhibited for cells pretreated with 1 μg/mL AMD3100. Beyond a critical time in the static culture, there is a gradual signal dropoff to the baseline level, similar to that under no CXCL12 stimulation. This is attributed to CXCL12 diffusional limitations, since this is not apparent in the dynamic 3D culture that constantly is replenished with CXCL12. When 66 ng/mL CXCL12 is used in the medium under dynamic 3D culture (7.5 μL/min perfusion) to create a gradient across the width of the cell-laden hydrogel, the fluorescence signal (Figures 4B(i) and S3) develops rapidly at proximal channel widths (0.25 mm) while taking longer to extend to 1 and 1.5 mm widths. With cells pretreated with 1 μg/mL AMD3100, the fluorescence level is diminished at the same widths (Figure 4B(ii)). Comparison of the normalized fluorescence signal with CXCL12 shows a 1.8-fold increase over the control, while the level remains close to the control after inhibitor pretreatment (Figure 4B(iii)). This validates ability of the open-top microfluidic system to deliver well-controlled cues from the CXC12 gradient to cells across the hydrogel width and cause its inhibition with AMD3100.

## Conclusions and Outlook

We present patterning of open-top 3D cultures that are integrated with microfluidic flow control to deliver biochemical cues, such as chemotactic gradients, for the purpose of assaying dynamic cell responses, such as cell migration. This open-top microfluidic approach using longitudinal aspiration and injection flows over 15 mm of the hydrogel/fluidic channel boundary, creates a sustained gradient across the hydrogel width (2 mm) that extends invariantly over the entire hydrogel depth (1 mm). This long and continuous hydrogel to fluidic channel interface is used to pattern the chemotactic gradient and sustain glioma cells over the 48 h culture period, whereas the posts used in prior reports within closed microfluidic systems create discontinuous interfaces, while challenges to maintaining a high aspect ratio limit the hydrogel depth to <0.1 mm. A GelMA–NorHA hybrid hydrogel was used to support both glioma cell viability and migration. The improved cytocompatibility of glioma cells in NorHA versus GelMA hydrogels is attributed to their rapid cross-linking ability due to the lower free radical levels needed to initiate step-growth polymerization, whereas the GelMA hydrogels formed by chain-growth cross-linking require a higher concentration of free radicals for polymerization initiation, leading to a loss in viability. Formation of chemotactic gradients was quantified using FITC-dextran of different molecular weights. The effect of chemotactic gradients across the hydrogel width on glioma cell responses, such as migration, was quantified by the fluorescence signal due to CXCL12, with signal inhibition through AMD3100 pretreatment. Improvements in spatial resolution would enable accurate determination of the specific hydrogel width regions that present significant differences in migration cues for modeling brain microenvironments. This open-top hydrogel microfluidic system requires release of the PDMS master mold from the cell-laden hydrogel for patterning adjoining fluidic channels. While release of the flexible silicone-like material is less likely to disrupt the adjoining UV-cross-linked cell-laden hydrogel pattern, inappropriate release can deform edges of the hydrogel region that interfaces with the fluid, thereby disrupting the ability to form long (∼cm scale) uninterrupted and continuous interfaces between the fluid and hydrogel. Hence, we optimized the UV cross-linking time to create well-cured hydrogels, while fixing the UV intensity at levels that maintain cell viability. Furthermore, the length to width (∼2 mm) and length to height (∼1 mm) aspect ratios for the hydrogel and channel patterns were maintained at 5 or less to ensure reproducible release of the PDMS master mold, without deformation to the edges of the hydrogel region that interfaces with the fluid. Given the challenges that closed microfluidic systems present to biocompatibility and growth factor loss from molecular adsorption, this open-top microfluidic system with longitudinal aspiration and injection flows can serve as an alternative platform for creating transverse chemotactic gradients across the 3D culture width, while extending invariantly over its length (∼15 mm) and depth (∼1 mm). This ability to create 3D cultures and chemotactic gradients over large lateral areas of approximately millimeter-scale depth that resemble the tissue microenvironment will be essential for *in vitro* disease modeling and emerging drug screening assays.

## Methods

### Materials

HA with a 20–28% degree of norbornene functionalization was synthesized by the Caliari group.^46^ LAP (Sigma), DTT (Sigma), and GelMA (300 g, 60%, Sigma) were used for the patterning of the hydrogel. For the temporal quantification of developed gradients, 0.33 mg/mL solutions of 10 kDa and 400 Da FITC-dextran (Sigma) were used. To study the migration response of U87 cells, cells were labeled with Fluo-4-AM (Thermo Fisher) and a CXCL12 (Biolegend) gradient in the presence or absence of pretreatment with AMD3100 (Sigma). 3-(Trimethoxysilyl)propyl methacrylate (Sigma) was used for surface modification of the glass slide.

### Cell Culture

U87 cells were cultured in 1× MEM (Gibco) supplemented with 10% FBS (Gibco) and pen-strep (100 units/mL penicillin + 100 μg/mL streptavidin) at 37 °C in a humidified incubator. For harvest upon confluency (80%), the medium was removed, and cells were washed in 1× PBS (Thermo Fisher), followed by a 0.25% trypsin-EDTA treatment (Gibco) for 5 min, after which complete medium was added. Cells were then pelleted at 300*g* for 10 min. For migration assays, cells were loaded with Fluo-4-AM dye (2.5 μM), and CXCL12 (66 ng/mL) was added to the dynamic 3D culture to generate a gradient across the cell-laden hydrogel, containing cells without or with AMD3100 pretreatment^54^ (1 μg/mL at 37 °C for 30 min).

### Hydrogel Composition

For experiments in which cells were encapsulated in the hybrid hydrogel, ∼2–3 × 10^6^ cells were pelleted and resuspended in 100–200 μL of 1× PBS. Hydrogel precursor solution was added to the cell solution, such that the final solution contained 2% GelMA, 1% NorHA, 0.23 mg/mL DTT, and 0.0328 wt % LAP and the U87 cells at a concentration of ∼4 × 10^6^ cells/ml in 1× PBS.

### Nanoindentation

A displacement-controlled nanoindenter (Optics 11 Piuma) was utilized to measure the elastic modulus of the photo-cross-linked hydrogel. After calibration of the nanoindenter, a hydrogel sample was loaded. Three measurements were taken for each hydrogel. An array of indentations were made at each measurement site to collect the data necessary for the analysis. Using the Hertzian contact mechanics model and assuming a Poission’s ratio of 0.5, the Young’s modulus was determined through the loading portion of the force versus distance indentation curve generated by the nanoindenter software. The elasticity data were analyzed and plotted using MATLAB.

### 3D Printing for Sample Holder and PDMS Molding

To pattern the hydrogel on the glass slide, a temporary PDMS mold was used. This mold was made using soft lithography techniques by casting 10:1 PDMS (Dow) on a 3D-printed master mold overnight at 60 C. The 3D-printed master mold was designed such that the patterned hydrogel and fluidic channel would have a total depth of ∼1 mm, the fluidic channel would have a width of 2 mm and be ∼15 mm long, and the hydrogel would be 2 mm wide in the center and have a total length of ∼22 mm. The PDMS master mold was printed using a Cadworks3D PR series printer in a Master Mold for PDMS Resin. The 3D-printed holder for interfacing the patterned hydrogel with fluidics was printed in Clear Microfluidics Resin (Cadworks3D) to enable transmitted light imaging. The holder was designed to have two holes on each side, with the same diameter as 1/16 in. microfluidic tubing through which the tubing is threaded, to serve as ports for the injection and aspiration. The holder was machined to rest on the edge of the Petri dish, with the ports designed such that the injection tubing can deliver fluid directly into the channel, while the aspiration tubing can be set to the height of the hydrogel.

### Open-Top Hydrogel Patterning

Glass slides were methacrylate-silanized using 5 mL of 1:100 3-(trimethoxysilyl)propyl methacrylate in ethanol, with 150 μL of 1:10 diluted glacial acetic acid in water added and mixed in. This solution was pipetted onto fully coated plasma-cleaned glass slides (Tergeo Plasma Cleaner) and left for 3 h at room temperature. Glass slides were then rinsed three times in ethanol, followed by three rinses in distilled water. Slides were allowed to dry and were sterilized under a UV lamp for 6 h. Cured PDMS was detached from the 3D-printed PDMS mold carefully, and an inlet and outlet were drilled using a biopsy punch, followed by cleaning of the PDMS with compressed nitrogen to remove any dust, and rinsing of the PDMS mold in water. The PDMS molds were then sterilized under a UV lamp for 6 h. Prior to patterning, the PDMS mold was immersed in a 2% BSA solution (in 1× PBS) for 45 min, after which the PDMS mold was allowed to air-dry. The PDMS mold was brought into conformal contact with the silanized glass slide, leading to a reversible bond. Through the inlet, a cell-laden hydrogel precursor solution was filled into the mold. Photopolymerization of the hydrogel was carried out using 365 nm UV light at 5 mW/cm^2^ for 120 s (Omnicure S2000). After 3 min, the PDMS mold was gently peeled away from the glass slide, with the patterned hydrogel adhered to the glass. The cell-laden hydrogel was carefully washed in 1× PBS, and complete medium was added such that the medium level was in line with the top surface of the hydrogel.

### Fluidic Interfacing to Open-Top Patterned Hydrogels

The patterned hydrogel was moved to a microscope stage, and the 3D-printed fluidic holder was placed on top of it, with injection tubing to deliver fluid into the fluidic channel and aspiration tubing set on the top surface of the hydrogel. Two channels of an MFCS-EZ pump (Fluigent) were used for fluid injection: one was connected to a reservoir containing culture medium or 1× PBS, while the other was connected to a reservoir containing either FITC-dextran (for profiles in Figure 3) or CXCL12, in the absence or presence of AMD3100 (for profiles in Figure 4). Tubing from the injection reservoirs was connected to Flow-EZ flow sensors (Fluigent) and set up using the control software to deliver a continuous steady flow rate of 7.5 μL/min to the hydrogel channels. Tubing was routed from the flow sensors through the on-stage incubator into the hydrogel through the 3D-printed holder. Aspiration tubing from the 3D-printed holder was connected to a reservoir that was under negative pressure using a vacuum pump (Cellix). Vacuum levels were modulated by using an in-line air valve. All microfluidic connections were made using 1/16 in. outer diameter microfluidic tubing.

### Time-Lapse Imaging

*Z*-stack time-lapse images through the depth of the hydrogel were acquired using an EVOS 620 microscope at 10× magnification with an on-stage incubator (ThermoFisher) every 15 min for 24 h. The on-stage incubator was set to maintain a humidified environment at 37 °C, with 5% CO_2_ for maintaining cell viability. Microfluidic tubing was routed through the machined holes on the on-stage incubator for delivery of medium and chemoattractant, as well as for aspiration.

### Image Analysis

ImageJ analysis was performed to capture the fluorescence signal from FITC-dextran and from cells cultured in monoculture, as well as cells encapsulated in 3D hydrogels. The grayscale intensity levels of individual cells were measured in each field of view to calculate the change in signal intensity over time due to stimulation. The distance from the hydrogel channel was measured based on the scale of the captured images. Cells were measured within similar corresponding distances from the channel boundary to quantify the spatiotemporal signal gradient. All signal intensities were normalized to a common control and plotted by utilizing MATLAB. For quantification of migration velocity in the hydrogels, migrating cells were identified at random and tracked from an origin point. Based on the scale, the distances were measured between each time point. The velocities between each time point were averaged over the duration of the experiment and plotted using MATLAB.

### Statistical Analysis

Statistical analysis was performed in MATLAB and presented as the respective mean ± standard deviation for each data point in Figures 2–4, based on at least three experiments conducted on the hydrogel-integrated fluidic system. Significance was calculated by one-way ANOVA with Tukey’s post-hoc test, with *p* ≤ 0.05 considered as significant.

## Supporting Information Available

Confirmation of viability of a 3D culture of highly migratory and malignant oligodendrocyte progenitor cells (Figure S1), fluorescence depth and width profiles with 10 kDa FITC-Dextran (Figure S2), and raw images of calcium ion influx into U87 cells under a CXCL12 gradient due to chemoattractant binding to its receptor (PDF)
Movie S1 showing the migration activity of U87 cells in the hybrid hydrogel (MOV)
Movie S2 showing the temporal evolution of the concentration gradients of 10 000 Da FITC-dextran across the hydrogel width (MOV)

## Supplementary Material

ab4c00041_si_001.pdf

ab4c00041_si_002.mov

ab4c00041_si_003.mov

## References

[ref1] Ostrom Q. T.; Gittleman H.; Truitt G.; Boscia A.; Kruchko C.; Barnholtz-Sloan J. S. CBTRUS statistical report: primary brain and other central nervous system tumors diagnosed in the United States in 2011–2015. Neuro-oncology 2018, 20 (suppl_4), iv1–iv86. 10.1093/neuonc/noy131. 30445539 PMC6129949

[ref2] Chaichana K. L.; Zadnik P.; Weingart J. D.; Olivi A.; Gallia G. L.; Blakeley J.; Lim M.; Brem H.; Quiñones-Hinojosa A. Multiple resections for patients with glioblastoma: prolonging survival. J. Neurosurg. 2013, 118 (4), 812–820. 10.3171/2012.9.JNS1277. 23082884 PMC3700339

[ref3] Wong B. S.; Shah S. R.; Yankaskas C. L.; Bajpai V. K.; Wu P.-H.; Chin D.; Ifemembi B.; ReFaey K.; Schiapparelli P.; Zheng X.; et al. A microfluidic cell-migration assay for the prediction of progression-free survival and recurrence time of patients with glioblastoma. Nat. Biomed. Eng. 2021, 5 (1), 26–40. 10.1038/s41551-020-00621-9. 32989283 PMC7855796

[ref4] Logun M.; Zhao W.; Mao L.; Karumbaiah L. Microfluidics in malignant glioma research and precision medicine. Adv. Biosyst. 2018, 2 (5), 1700221 10.1002/adbi.201700221. 29780878 PMC5959050

[ref5] Pedron S.; Becka E.; Harley B. A. Regulation of glioma cell phenotype in 3D matrices by hyaluronic acid. Biomaterials 2013, 34 (30), 7408–7417. 10.1016/j.biomaterials.2013.06.024. 23827186

[ref6] Giarra S.; Ierano C.; Biondi M.; Napolitano M.; Campani V.; Pacelli R.; Scala S.; De Rosa G.; Mayol L. Engineering of thermoresponsive gels as a fake metastatic niche. Carbohydr. Polym. 2018, 191, 112–118. 10.1016/j.carbpol.2018.03.016. 29661298

[ref7] Kasapidou P. M.; de Montullé E. L.; Dembélé K.-P.; Mutel A.; Desrues L.; Gubala V.; Castel H. Hyaluronic acid-based hydrogels loaded with chemoattractant and anticancer drug–new formulation for attracting and tackling glioma cells. Soft Matter 2021, 17 (48), 10846–10861. 10.1039/D1SM01003D. 34806746

[ref8] Young E. W.; Beebe D. J. Fundamentals of microfluidic cell culture in controlled microenvironments. Chem. Soc. Rev. 2010, 39 (3), 1036–1048. 10.1039/b909900j. 20179823 PMC2967183

[ref9] Varma S.; Voldman J. Caring for cells in microsystems: principles and practices of cell-safe device design and operation. Lab Chip 2018, 18 (22), 3333–3352. 10.1039/C8LC00746B. 30324208 PMC6254237

[ref10] Monteduro A. G.; Rizzato S.; Caragnano G.; Trapani A.; Giannelli G.; Maruccio G. Organs-on-chips technologies – A guide from disease models to opportunities for drug development. Biosens. Bioelectron. 2023, 231, 115271 10.1016/j.bios.2023.115271. 37060819

[ref11] Carter S.-S. D.; Atif A.-R.; Kadekar S.; Lanekoff I.; Engqvist H.; Varghese O. P.; Tenje M.; Mestres G. PDMS leaching and its implications for on-chip studies focusing on bone regeneration applications. Organs-on-a-Chip 2020, 2, 100004 10.1016/j.ooc.2020.100004.

[ref12] Castiaux A. D.; Spence D. M.; Martin R. S. Review of 3D Cell Culture with Analysis in Microfluidic Systems. Anal. Methods 2019, 11 (33), 4220–4232. 10.1039/C9AY01328H. 32051693 PMC7015157

[ref13] van Meer B. J.; de Vries H.; Firth K. S. A.; van Weerd J.; Tertoolen L. G. J.; Karperien H. B. J.; Jonkheijm P.; Denning C.; Ijzerman A. P.; Mummery C. L. Small molecule absorption by PDMS in the context of drug response bioassays. Biochem. Biophys. Res. Commun. 2017, 482 (2), 323–328. 10.1016/j.bbrc.2016.11.062. 27856254 PMC5240851

[ref14] Toepke M. W.; Beebe D. J. PDMS absorption of small molecules and consequences in microfluidic applications. Lab Chip 2006, 6 (12), 1484–1486. 10.1039/b612140c. 17203151

[ref15] Markov D. A.; Lillie E. M.; Garbett S. P.; McCawley L. J. Variation in diffusion of gases through PDMS due to plasma surface treatment and storage conditions. Biomed. Microdevices 2014, 16 (1), 91–6. 10.1007/s10544-013-9808-2. 24065585 PMC3945670

[ref16] Heo Y. S.; Cabrera L. M.; Song J. W.; Futai N.; Tung Y.-C.; Smith G. D.; Takayama S. Characterization and Resolution of Evaporation-Mediated Osmolality Shifts That Constrain Microfluidic Cell Culture in Poly(dimethylsiloxane) Devices. Anal. Chem. 2007, 79 (3), 1126–1134. 10.1021/ac061990v. 17263345 PMC2605290

[ref17] Hosic S.; Bindas A. J.; Puzan M. L.; Lake W.; Soucy J. R.; Zhou F.; Koppes R. A.; Breault D. T.; Murthy S. K.; Koppes A. N. Rapid Prototyping of Multilayer Microphysiological Systems. ACS Biomater. Sci. Eng. 2021, 7 (7), 2949–2963. 10.1021/acsbiomaterials.0c00190. 34275297 PMC8290094

[ref18] Torino S.; Corrado B.; Iodice M.; Coppola G. PDMS-Based Microfluidic Devices for Cell Culture. Inventions 2018, 3 (3), 65 10.3390/inventions3030065.

[ref19] Sackmann E. K.; Fulton A. L.; Beebe D. J. The present and future role of microfluidics in biomedical research. Nature 2014, 507 (7491), 181–189. 10.1038/nature13118. 24622198

[ref20] Cao U. M. N.; Zhang Y.; Chen J.; Sayson D.; Pillai S.; Tran S. D. Microfluidic Organ-on-A-chip: A Guide to Biomaterial Choice and Fabrication. Int. J. Mol. Sci. 2023, 24 (4), 3232 10.3390/ijms24043232. 36834645 PMC9966054

[ref21] Lee U. N.; Day J. H.; Haack A. J.; Bretherton R. C.; Lu W.; DeForest C. A.; Theberge A. B.; Berthier E. Layer-by-layer fabrication of 3D hydrogel structures using open microfluidics. Lab Chip 2020, 20 (3), 525–536. 10.1039/C9LC00621D. 31915779 PMC8018606

[ref22] Su C.; Chuah Y. J.; Ong H. B.; Tay H. M.; Dalan R.; Hou H. W. A Facile and Scalable Hydrogel Patterning Method for Microfluidic 3D Cell Culture and Spheroid-in-Gel Culture Array. Biosensors 2021, 11 (12), 509 10.3390/bios11120509. 34940266 PMC8699815

[ref23] Pei J.; Sun Q.; Yi Z.; Li Q.; Wang X. Recoverable elastic barrier for robust hydrogel patterning with uniform flow profile for organ-on-a-chip applications. J. Micromech. Microeng. 2020, 30 (3), 035005 10.1088/1361-6439/ab68b2.

[ref24] Menon N. V.; Tay H. M.; Wee S. N.; Li K. H. H.; Hou H. W. Micro-engineered perfusable 3D vasculatures for cardiovascular diseases. Lab Chip 2017, 17 (17), 2960–2968. 10.1039/C7LC00607A. 28740980

[ref25] Trietsch S. J.; Israëls G. D.; Joore J.; Hankemeier T.; Vulto P. Microfluidic titer plate for stratified 3D cell culture. Lab Chip 2013, 13 (18), 3548–3554. 10.1039/c3lc50210d. 23887749

[ref26] Varhue W. B.; Rane A.; Castellanos-Sanchez R.; Peirce S. M.; Christ G.; Swami N. S. Perfusable cell-laden micropatterned hydrogels for delivery of spatiotemporal vascular-like cues to tissues. Organs-on-a-Chip 2022, 4, 100017 10.1016/j.ooc.2022.100017. 36865345 PMC9977322

[ref27] Su C.; Menon N. V.; Xu X.; Teo Y. R.; Cao H.; Dalan R.; Tay C. Y.; Hou H. W. A novel human arterial wall-on-a-chip to study endothelial inflammation and vascular smooth muscle cell migration in early atherosclerosis. Lab Chip 2021, 21 (12), 2359–2371. 10.1039/D1LC00131K. 33978037

[ref28] Park D.; Lee J.; Lee Y.; Son K.; Choi J. W.; Jeang W. J.; Choi H.; Hwang Y.; Kim H.-Y.; Jeon N. L. Aspiration-mediated hydrogel micropatterning using rail-based open microfluidic devices for high-throughput 3D cell culture. Sci. Rep. 2021, 11 (1), 19986 10.1038/s41598-021-99387-6. 34620916 PMC8497476

[ref29] Park D.; Son K.; Hwang Y.; Ko J.; Lee Y.; Doh J.; Jeon N. L. High-Throughput Microfluidic 3D Cytotoxicity Assay for Cancer Immunotherapy (CACI-IMPACT Platform). Front. Immunol. 2019, 10, 1133 10.3389/fimmu.2019.01133. 31191524 PMC6546835

[ref30] Qasaimeh M. A.; Ricoult S. G.; Juncker D. Microfluidic probes for use in life sciences and medicine. Lab Chip 2013, 13 (1), 40–50. 10.1039/C2LC40898H. 23042577

[ref31] Brimmo A.; Goyette P.-A.; Alnemari R.; Gervais T.; Qasaimeh M. A. 3D Printed Microfluidic Probes. Sci. Rep. 2018, 8 (1), 10995 10.1038/s41598-018-29304-x. 30030464 PMC6054653

[ref32] Qasaimeh M. A.; Pyzik M.; Astolfi M.; Vidal S. M.; Juncker D. Neutrophil Chemotaxis in Moving Gradients. Adv. Biosyst. 2018, 2 (7), 1700243 10.1002/adbi.201700243.

[ref33] Juncker D.; Schmid H.; Delamarche E. Multipurpose microfluidic probe. Nat. Mater. 2005, 4 (8), 622–628. 10.1038/nmat1435. 16041377

[ref34] Cors J. F.; Lovchik R. D.; Delamarche E.; Kaigala G. V. A compact and versatile microfluidic probe for local processing of tissue sections and biological specimens. Rev. Sci. Instrum. 2014, 85 (3), 034301 10.1063/1.4866976. 24689601

[ref35] Shinha K.; Nihei W.; Kimura H. A Microfluidic Probe Integrated Device for Spatiotemporal 3D Chemical Stimulation in Cells. Micromachines 2020, 11 (7), 691 10.3390/mi11070691. 32708814 PMC7408473

[ref36] Zhang Q.; Mao S.; Khan M.; Feng S.; Zhang W.; Li W.; Lin J.-M. In Situ Partial Treatment of Single Cells by Laminar Flow in the “Open Space. Anal. Chem. 2019, 91 (2), 1644–1650. 10.1021/acs.analchem.8b05313. 30558412

[ref37] Lovchik R. D.; Kaigala G. V.; Georgiadis M.; Delamarche E. Micro-immunohistochemistry using a microfluidic probe. Lab Chip 2012, 12 (6), 1040–1043. 10.1039/c2lc21016a. 22237742

[ref38] Queval A.; Ghattamaneni N. R.; Perrault C. M.; Gill R.; Mirzaei M.; McKinney R. A.; Juncker D. Chamber and microfluidic probe for microperfusion of organotypic brain slices. Lab Chip 2010, 10 (3), 326–334. 10.1039/B916669F. 20091004

[ref39] Qasaimeh M. A.; Gervais T.; Juncker D. Microfluidic quadrupole and floating concentration gradient. Nat. Commun. 2011, 2 (1), 464 10.1038/ncomms1471. 21897375 PMC3984239

[ref40] Safavieh M.; Qasaimeh M. A.; Vakil A.; Juncker D.; Gervais T. Two-Aperture Microfluidic Probes as Flow Dipoles: Theory and Applications. Sci. Rep. 2015, 5 (1), 11943 10.1038/srep11943. 26169160 PMC4500946

[ref41] Xie L.; Kang H.; Xu Q.; Chen M. J.; Liao Y.; Thiyagarajan M.; O’Donnell J.; Christensen D. J.; Nicholson C.; Iliff J. J.; Takano T.; et al. Sleep drives metabolite clearance from the adult brain. Science 2013, 342 (6156), 373–377. 10.1126/science.1241224. 24136970 PMC3880190

[ref42] Ray L. A.; Heys J. J. Fluid flow and mass transport in brain tissue. Fluids 2019, 4 (4), 196 10.3390/fluids4040196.

[ref43] Groothuis D. R.; Vavra M. W.; Schlageter K. E.; Kang E. W.; Itskovich A. C.; Hertzler S.; Allen C. V.; Lipton H. L. Efflux of drugs and solutes from brain: the interactive roles of diffusional transcapillary transport, bulk flow and capillary transporters. J. Cereb. Blood Flow Metab. 2007, 27 (1), 43–56. 10.1038/sj.jcbfm.9600315. 16639426

[ref44] Goodarzi K.; Rao S. S. Hyaluronic acid-based hydrogels to study cancer cell behaviors. J. Mater. Chem. B 2021, 9 (31), 6103–6115. 10.1039/D1TB00963J. 34259709

[ref45] Yue K.; Trujillo-de Santiago G.; Alvarez M. M.; Tamayol A.; Annabi N.; Khademhosseini A. Synthesis, properties, and biomedical applications of gelatin methacryloyl (GelMA) hydrogels. Biomaterials 2015, 73, 254–71. 10.1016/j.biomaterials.2015.08.045. 26414409 PMC4610009

[ref46] Unal D. B.; Caliari S. R.; Lampe K. J. 3D Hyaluronic Acid Hydrogels for Modeling Oligodendrocyte Progenitor Cell Behavior as a Function of Matrix Stiffness. Biomacromolecules 2020, 21 (12), 4962–4971. 10.1021/acs.biomac.0c01164. 33112592

[ref47] Budday S.; Nay R.; de Rooij R.; Steinmann P.; Wyrobek T.; Ovaert T. C.; Kuhl E. Mechanical properties of gray and white matter brain tissue by indentation. J. Mech. Behav. Biomed. Mater. 2015, 46, 318–30. 10.1016/j.jmbbm.2015.02.024. 25819199 PMC4395547

[ref48] Vigata M.; Meinert C.; Bock N.; Dargaville B. L.; Hutmacher D. W. Deciphering the molecular mechanism of water interaction with gelatin methacryloyl hydrogels: Role of ionic strength, ph, drug loading and hydrogel network characteristics. Biomedicines 2021, 9 (5), 574 10.3390/biomedicines9050574. 34069533 PMC8161260

[ref49] Lin C. C.; Sawicki S. M.; Metters A. T. Free-Radical-Mediated Protein Inactivation and Recovery during Protein Photoencapsulation. Biomacromolecules 2008, 9 (1), 75–83. 10.1021/bm700782c. 18088094

[ref50] Fairbanks B. D.; Schwartz M. P.; Halevi A. E.; Nuttelman C. R.; Bowman C. N.; Anseth K. S. A Versatile Synthetic Extracellular Matrix Mimic via Thiol-Norbornene Photopolymerization. Adv. Mater. 2009, 21 (48), 5005–5010. 10.1002/adma.200901808. 25377720 PMC4226179

[ref51] Mũnoz Z.; Shih H.; Lin C. C. Gelatin hydrogels formed by orthogonal thiol–norbornene photochemistry for cell encapsulation. Biomater. Sci. 2014, 2 (8), 1063–1072. 10.1039/C4BM00070F. 32482001

[ref52] Liu C.; Sage J. C.; Miller M. R.; Verhaak R. G. W.; Hippenmeyer S.; Vogel H.; Foreman O.; Bronson R. T.; Nishiyama A.; Luo L.; Zong H. Mosaic Analysis with Double Markers Reveals Tumor Cell-of-Origin in Glioma. Cell 2011, 146, 209–221. 10.1016/j.cell.2011.06.014. 21737130 PMC3143261

[ref53] Ledur P.; Liu C.; He H.; Harris A.; Minussi D.; Zhou H.; Shaffrey M.; Asthagiri A.; Lopes M.; Schiff D.; Lu Y.; Mandell J.; Lenz G.; Zong H. Culture conditions tailored to the cell of origin are critical for maintaining native properties and tumorigenicity of glioma cells. Neuro-Oncology 2016, 18 (10), 1413–1424. 10.1093/neuonc/now062. 27106408 PMC5035523

[ref54] Hatse S.; Princen K.; Bridger G.; De Clercq E.; Schols D. Chemokine receptor inhibition by AMD3100 is strictly confined to CXCR4. FEBS Lett. 2002, 527 (1–3), 255–262. 10.1016/S0014-5793(02)03143-5. 12220670

