# Supplementary material for: Open-Top Patterned Hydrogel-Laden 3D Glioma Cell Cultures for Creation of Dynamic Chemotactic Gradients to Direct Cell Migration
Source: ACS Biomater Sci Eng. 2024 Apr 23;10(5):3470–7. doi: 10.1021/acsbiomaterials.4c00041 (PMC11094679; doi:10.1021/acsbiomaterials.4c00041)
Supplement: Supplementary file 1 — ab4c00041_si_001.pdf [file ab4c00041_si_001.pdf]

Open-top patterned hydrogel-laden 3D glioma cell cultures for creation of dynamic chemotactic gradients to direct cell migration

Aditya Rane[a,#], Steven Tate[b,#], Jenna L. Sumey[c], Qing Zhong[d], Hui Zong[e], Benjamin Purow[d], Steven Caliari[c,f], Nathan S. Swami [a,b,*]

a – Chemistry, University of Virginia, Charlottesville, Virginia 22904, USA;

b – Electrical and Computer Engineering, University of Virginia, Charlottesville, Virginia 22904, USA;

c – Chemical Engineering, University of Virginia, Charlottesville, Virginia 22904, USA;

d – Neurology, School of Medicine, University of Virginia, Charlottesville, VA 22903;

e – Microbiology, Immunology & Cancer Biology, School of Medicine, University of Virginia, Charlottesville, VA 22903.

f – Biomedical Engineering, University of Virginia, Charlottesville, VA 22904, USA;

Supplementary Results

Figure S1. The hybrid hydrogel described in **Figure 2** of the manuscript supports viability of a 3D culture of the highly migratory and malignant oligodendrocyte progenitor cells (OPCs with GFP expression of 19222 and 10301 from two different mouse models) that are progenitors of glioma. Based on viable OPCs that continue to exhibit GFP expression, the viability loss is ~15-20% at 12 h.

Figure S2. Fluorescence images across patterned hydrogel width at 24 h timepoint (with 10,000 Da FITC-dextran in channel at edge of hydrogel at width labeled as 0 mm), shown in (i-iii) at various depth levels of the hydrogel show steady-state levels at widths > 1 mm and similarity of the fluorescence profiles across the 1 mm hydrogel depth.

Figure S3. i. U87 cells in the 3D hydrogel culture under a gradient of CXCL12 exhibit calcium ion influx into the cell upon binding of the chemoattractant to its receptor on the cell membrane, thereby causing glioma cells labeled with a calcium signaling probe to exhibit higher fluorescence levels upon calcium binding. This is used in Fig. 4B(i) to visualize the effect of the chemotactic gradient on cells across the hydrogel width. **ii.** Representative images of cells in hydrogel showing no fluorescence initially, at t = 15 minutes; and **iii.** higher fluorescence in cells at t = 225 mins.

Movie S1. shows the migration activity of U87 cells (no chemotactic gradient) after 48 h of 3D culture in the hybrid hydrogel described in Figure 2 of the manuscript, whereas NorHA gels show minimal to no cell migration.

Movie S2. Temporal evolution of the concentration gradients of 10,000 Da FITC-dextran across the hydrogel width over 12 h shown in **Figure 3B(ii)** of the manuscript.

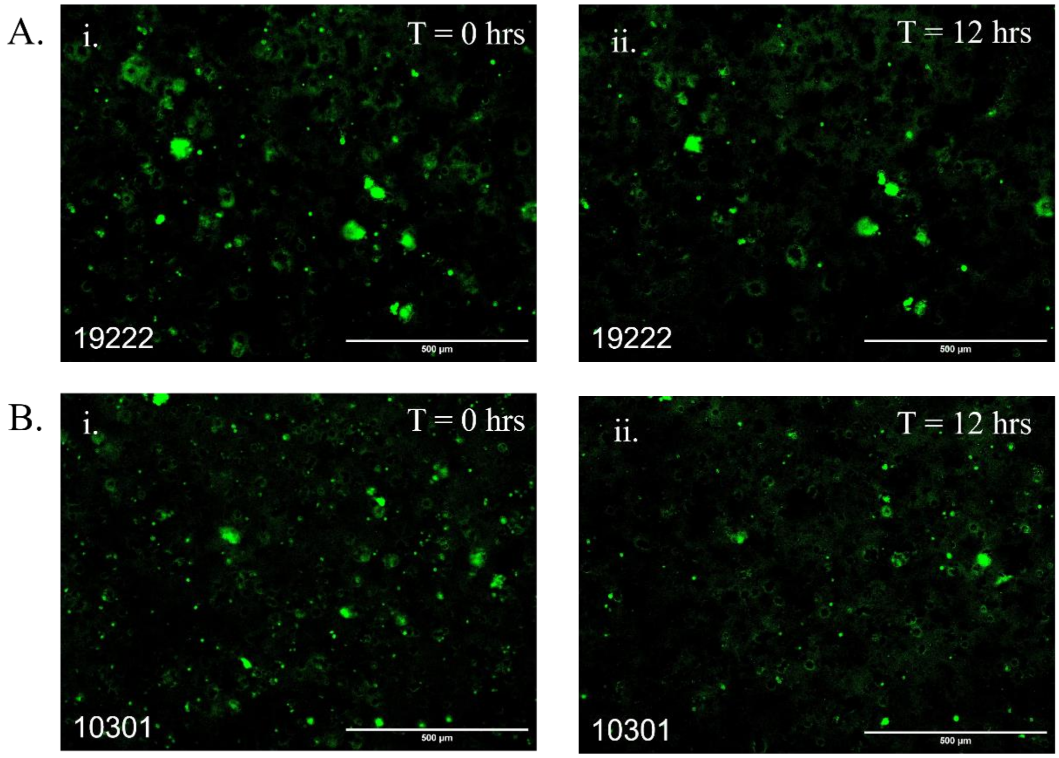

Figure S1. The hybrid hydrogel described in **Figure 2** of the manuscript supports viability of a 3D culture of the highly migratory and malignant oligodendrocyte progenitor cells (OPCs with GFP expression of 19222 and 10301 from two different mouse models) that are progenitors of glioma. Based on viable OPCs that continue to exhibit GFP expression, the viability loss is ~15-20% 0 hours (A. i & B. i) vs. at the 12 hour timepoint (A. ii & B.ii).

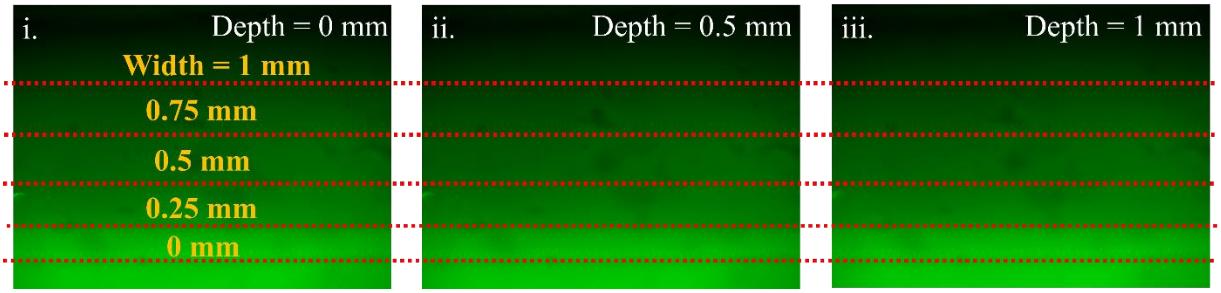

Figure S2. Similarity of the chemotactic gradient across the hydrogel depth based on fluorescence images across patterned hydrogel width acquired at 24 h timepoint across depth levels of: (i) 0 mm; (ii) 0.5 mm; and (iii) 1 mm. Images based on 10,000 Da FITC-dextran in channel at edge of hydrogel at width labeled as 0 mm create profiles across the hydrogel width (each dotted line indicates the respective width).

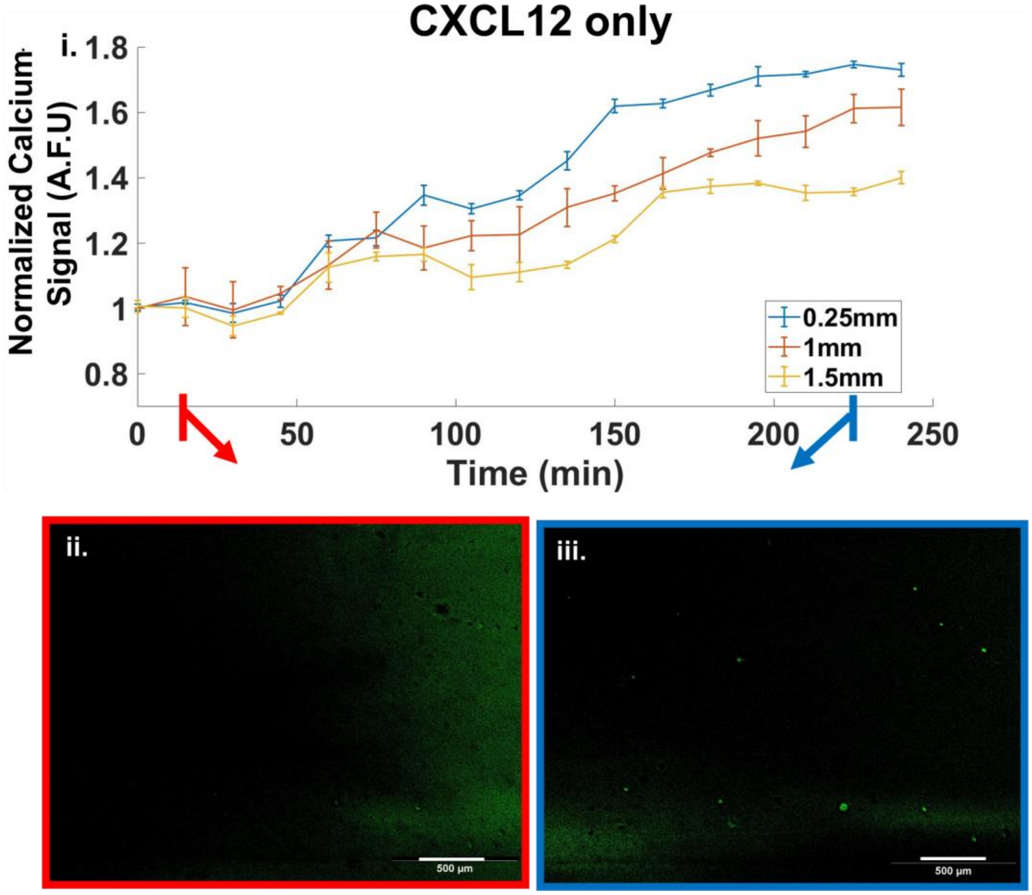

Figure S3. i. U87 cells in the 3D hydrogel culture under a gradient of CXCL12 exhibit calcium ion influx into the cell upon binding of the chemoattractant to its receptor on the cell membrane, thereby causing glioma cells labeled with a calcium signaling probe to exhibit higher fluorescence levels upon calcium binding. This is used in Fig. 4B(i) to visualize the effect of the chemotactic gradient on cells across the hydrogel width. **ii.** Representative images of cells in hydrogel (0.25 mm width) showing no fluorescence initially, at t = 15 minutes; and **iii.** higher fluorescence in cells at t = 225 mins.

Movie M1. shows the migration activity of U87 cells (no chemotactic gradient) after 48 h of 3D culture in the hybrid hydrogel described in Figure 2 of the manuscript, whereas NorHA gels show minimal to no cell migration.

Movie M2. Temporal evolution of the concentration gradients of 10,000 Da FITC-dextran across the hydrogel width over 12 h shown in **Figure 3B(ii)** of the manuscript.
